# The Functions of Mitochondrial 2′,3′-Cyclic Nucleotide-3′-Phosphodiesterase and Prospects for Its Future

**DOI:** 10.3390/ijms21093217

**Published:** 2020-05-01

**Authors:** Krestinina Olga, Baburina Yulia, Papadopoulos Vassilios

**Affiliations:** 1 Institute of Theoretical and Experimental Biophysics, Russian Academy of Sciences, Pushchino, 142290 Moscow region, Russia; byul@rambler.ru; 2 Department of Pharmacology and Pharmaceutical Sciences, School of Pharmacy, University of Southern California, Los Angeles, CA 90089, USA; vpapadop@usc.edu

**Keywords:** mitochondria, mitochondrial 2′,3′-cyclic nucleotide-3′-phosphodiesterase (mtCNPase), protein phosphorylation, aging, permeability transition pore (mPTP), heart failure

## Abstract

2′,3′-cyclic nucleotide-3′-phosphodiesterase (CNPase) is a myelin-associated enzyme that catalyzes the phosphodiester hydrolysis of 2’,3’-cyclic nucleotides to 2’-nucleotides. However, its presence is also found in unmyelinated cells and other cellular structures. Understanding of its specific physiological functions, particularly in unmyelinated cells, is still incomplete. This review concentrates on the role of mitochondrial CNPase (mtCNPase), independent of myelin. mtCNPase is able to regulate the functioning of the mitochondrial permeability transition pore (mPTP), and thus is involved in the mechanisms of cell death, both apoptosis and necrosis. Its participation in the development of various diseases and pathological conditions, such as aging, heart disease and alcohol dependence, is also reviewed. As such, mtCNPase can be considered as a potential target for the development of therapeutic strategies in the treatment of mitochondria-related diseases.

## 1. Introduction

In the central nervous system of mammals and some vertebrates, a myelin-associated enzyme 2′,3′-cyclic nucleotide 3′-phosphodiesterase (CNPase, EC3.1.4.37) is abundantly present. It is known that CNPase is able to catalyzes the hydrolysis of 2’,3’-cyclic nucleotides to produce 2’-nucleotides in vitro [1], but the physiologically substrate in vivo is still unclear. In addition, it was reported that the enzyme was present in a variety of other cell types, albeit at lower levels [2,3] and in non-myelin membrane preparations from the spleen, liver, thymus, adrenal glands, kidney, heart and skeletal muscle [4,5,6]. CNPase had also been observed to be associated with mitochondria in adrenal cells [7].

In oligodendrocytes, Schwann cells, and myelin from all species, there are two isoforms of CNPase (CNPase1—46 kDa, CNPase2—48 kDa) [8,9]. CNPase is encoded by a single gene located on chromosome 17q21.2. The gene consists of four exons and two promoters, giving rise to two RNA transcripts, one of which will generate the two proteins [10]. CNPase isoforms have identical amino acid sequences; however, CNPase2 has an additional 20 residues at the N-terminus [8,9]. These isoforms are enzymatically active and synthesized on free ribosomes [11]. At the C-terminus, both polypeptides are post-translationally modified by isoprenylation [12]. Stricker et al. found that CNPase1 was specifically phosphorylated by both protein kinase A and cGMP-dependent protein kinase [13]. Lee and co-authors demonstrated that, due to the mitochondrial targeting signal at the N-terminus, only CNPase2 was translocated to the mitochondria [14]. This importation was enabled through protein kinase C-mediated phosphorylation of the targeting signal. CNPase2 is completely treated in the adult liver and embryonic brain, displaying that it is localized specifically to mitochondria in non-myelinating cells. Thus, it was supposed that the biological role of CNPase2 in mitochondria relates to myelination [14]. Furthermore, it was demonstrated that CNPase2 is relegated to the inner mitochondrial membrane on the side facing the intermembrane space. In the developing rat brain of amoeboid microglial cells, CNPase was associated mostly with the plasma membrane, filopodial projections and mitochondria [15]. It was previously reported that CNPase was localized to both the inner and outer membranes of mitochondria in rat brain mitochondria (RBM), presumably occupying the intermembrane space between the two [16]. Next, we will discuss the function of CNPase in mitochondria from the point of view of our research and call the enzyme mtCNPase (mitochondrial 2′,3′-cyclic nucleotide 3′-phosphodiesterase).

## 2. Detection of mtCNPase

**Figure 1:**
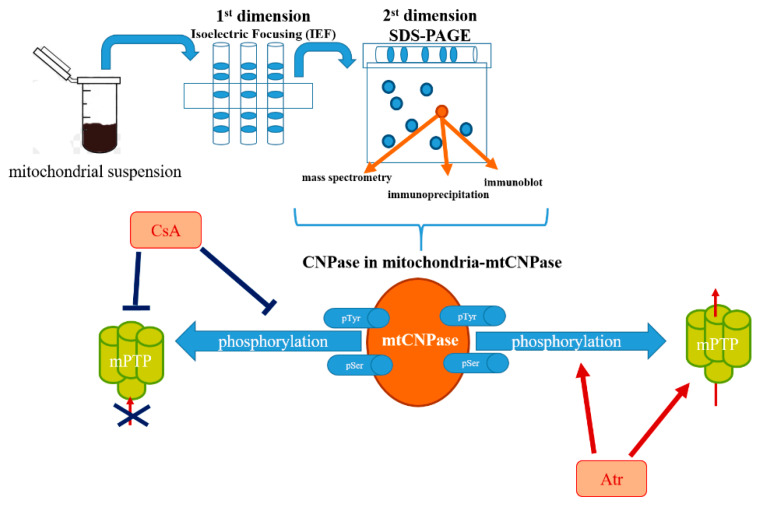
Schematic representation of the detection of mtCNPase in the mitochondria.

At first, we observed that there were proteins phosphorylated by a protein kinase in the presence of Ca^2+^ in RBM and rat liver mitochondria (RLM) [17,18,19,20]. Within the set of identified proteins, the most interesting had a molecular size of 46 kDa and its phosphorylation status was increased in Ca^2+^-overloaded RBM, i.e., when the mitochondrial permeability transition pore (mPTP) was opened. This 46 kDa phosphoprotein was identified as the phosphorylated form of CNPase by means of immunoblot, immunoprecipitation and mass spectrometry (Figure 1). CNPase1 or CNPase2 with its mitochondrial targeting domain already clipped off after it reached its cellular destination needs to be found out in further research. Moreover, we found that, in phosphorylated mitochondrial samples, this mtCNPase was immunoprecipitated both when the pore was closed and when it was opened; however, the phosphorylation status of mtCNPase was significantly higher with an mPTP opening. Phospho-Ser and phospho-Tyr levels were increased in mtCNPase immunoprecipitates that obtained from Ca^2+^-overloaded RBM. In RBM, this mtCNPase was located in the outer membrane and in mitoplasts [16]. No CNPase was found in the inter-membrane space. In our studies, the mtCNPase substrate 2′,3′-cAMP also accelerated mPTP opening, and the maximal efficiency of 2′,3′-cAMP-dependent mPTP activation was observed at 5–10 μM and was cyclosporin A (CsA)-sensitive. 2′,3′-cAMP was also able to induce high-amplitude, Ca^2+^-dependent swelling of the mitochondria. Moreover, it was demonstrated that 2′,3′-cAMP (5–10 μM) enhanced the level of mtCNPase phosphorylation in the presence of threshold Ca^2+^ concentrations [16].

Atractyloside was found to act as a noncompetitive inhibitor of CNPase activity [4,5] and also a stimulator of mPTP opening, stabilizing adenine nucleotide translocase (ANT) in the “c” state conformation, preferential for mPTP opening [21]. Atractyloside also stimulated the incorporation of ^32^P from [γ^−32^P]ATP into mtCNPase under conditions of mPTP opening. These findings suggest that compounds activating the mPTP opening have the ability to increase the phosphorylation of mtCNPase (46 kDa), indicating the connection between mPTP opening and mtCNPase phosphorylation. Interestingly, the endogenous expression of CNPase was highest in the oligodendrocyte cell line OLN93, and OLN93 cells transfected with siRNA targeting CNPase expressed reduced levels of CNPase. The diminished levels of CNPase were observed in mitochondria from OLN93 cells with CNPase knockdown that correlated with the facilitated activation of Ca^2+^ efflux from the mitochondria due to mPTP opening. This process was further stimulated in the presence of 2′,3′-cAMP [16]. The phosphorylated form of mtCNPase was found in mitochondria isolated from OLN93 cells, and the downregulation of mtCNPase expression led to reduced CNPase phosphorylation. The presence of the phosphorylated form of mtCNPase in mitochondria suggests that mtCNPase might participate in regulatory pathways in mitochondria that rely on phosphorylation [22].

## 3. Interaction Partners of mtCNPase

Dyer and co-authors showed that CNPase co-localized with both actin-based and tubulin-based cytoskeletons in cultured oligodendrocytes. [21]. Later, De Angelis and Braun found that CNPase actually bound to the actin-based cytoskeleton [23]. The additional biochemical evidence of CNPase–tubulin interaction was obtained from the observation that microtubules in cultured rat thyroid cells dissociated from the plasma membrane after treatment with lovastatin, a compound that inhibits isoprenylation. Since tubulin function does not require isoprenylation, this suggested that an isoprenylated linker protein must be responsible for the attachment to microtubule membranes. Such an isoprenylated protein with a molecular weight of 48 kDa was later identified as CNPase [24]. It was also found that CNPase not only associated with microtubules in cultured rat thyroid cells and brain tissue, but also co-purified with microtubules even after successive polymerization and depolymerization cycles. Thus, CNPase was identified as a microtubule-associated protein that also has microtubule polymerization activity in vitro [25].

mtCNPase was reported to be specifically associated with ADAP1, a brain-specific protein (known recently as p42^IP4^ or Centaurin-α1) and α-tubulin in RBM [26]. Interestingly, in the mitochondria, the associations of ADAP1 with mtCNPase, ADAP1 with α-tubulin, and mtCNPase with α-tubulin, were confirmed by co-immunoprecipitation experiments [26]. The co-immunoprecipitation can be taken as indication of in vivo interactions between the respective proteins. Importantly, it was revealed that the immunoprecipitate of ADAP1 from RBM contained immuno-reactive bands for both mtCNPase and α-tubulin antibodies. The immuno-reactive bands were not observed in immunoprecipitates obtained with myelin-based CNPase isoform control antibodies. The specificity of the co-immunoprecipitation of ADAP1 with α-tubulin and mtCNPase was confirmed by immunostaining with ANT antibody. Thus, the formation of an in vivo complex between ADAP1, mtCNPase and α-tubulin in RBM can be inferred. The involvement of ADAP1 and mtCNPase in Ca^2+^-induced mPTP opening was demonstrated independently in isolated mitochondria from various cells [16,26]. Therefore, it is of great interest to understand what the functional consequences are for the complex containing ADAP1, mtCNPase and α-tubulin formed in vivo in RBM.

**Figure 2:**
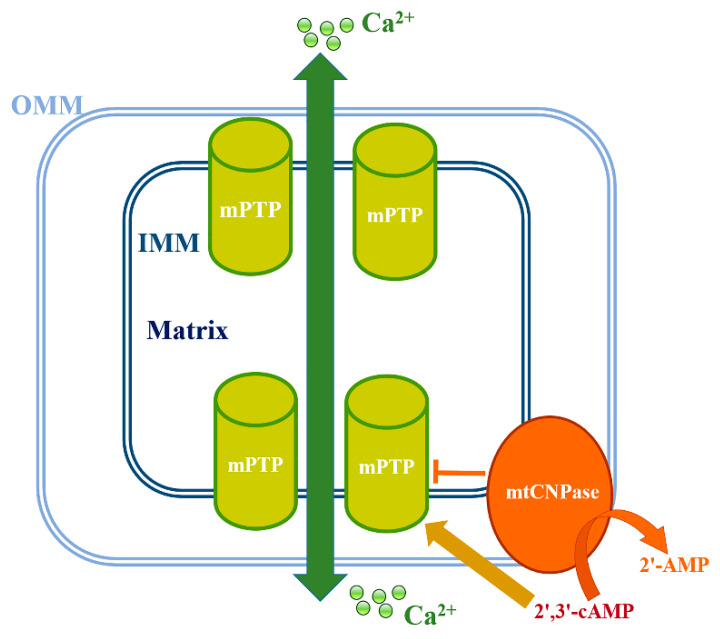
Schematic representation of the role of mtCNPase in mPTP function.

mtCNPase has been reported to be localized to both the inner and outer mitochondrial membranes, effectively placing it between them (Figure 2) [16]. This finding prompted us to look for mtCNPase -interacting proteins within contact sites, where the mPTP complex is located. ANT and the voltage-dependent anion channel (VDAC) were previously considered to be components of the mPTP complex. However, genetic studies have suggested that the composition of mPTP does not require VDAC and ANT [27,28], yet VDAC and ANT are still considered to be regulators/modulators of mPTP [29,30,31]. However, cyclophilin D (CyP-D) is a mitochondrial matrix protein considered as one of the critical elements for mPTP functioning [32]. It was shown that mtCNPase co-precipitated with basic mPTP regulators, such as CyP-D, VDAC and ANT [33]. Finding that mtCNPase co-localized with CyP-D, ANT and VDAC, as well as with α-tubulin in Ca^2+^-loaded and unloaded mitochondria, indicates the possible physical binding between these proteins in the mitochondria.

In the outer mitochondrial membrane, mtCNPase can interact with VDAC, which is the main outer membrane protein involved in the permeability of the outer mitochondrial membrane. VDAC can be in open or closed states. In the VDAC closed state, its channel is more permeable to Ca^2+^ [34], so that the acceleration of mPTP opening might result, and the binding of α-tubulin to VDAC facilitates its closure [35]. Since both VDAC and mtCNPase are bound to α-tubulin, VDAC conductance might be regulated directly by mtCNPase or through α-tubulin binding, permitting the modulation of the permeability of the outer membrane.

Co-precipitation of mtCNPase with CyP-D and ANT supported mtCNPase participation in the regulation of mPTP opening. It was also found that mtCNPase co-precipitated with cytochrome *c* oxidase IV (COX IV) [33]. Since mtCNPase is localized in mitoplasts, [16] along with the fact that mtCNPase co-localizes with COX IV, this indicates its possible interaction with respiratory chain functional complexes and ATP synthase in RBM. The effect of the inducer of mPTP, atractyloside, on mtCNPase association with complexes of respiratory chain and ATP synthase was also examined [33]. Atractyloside inhibits the transport of adenine nucleotides across the inner mitochondrial membrane by stabilizing ANT in the ′′c′′ state conformation that preferentially supports mPTP opening [36]. Atractyloside-sensitive nucleotide binding sites were discovered in both the inner and outer membranes in RLM [37]. It was observed that atractyloside facilitated mtCNPase association with complexes I and III by almost 100%, and the stimulated dissociation of mtCNPase from complex II and complex IV by 50%, whereas atractyloside only slightly the increased association of mtCNPase with the ATP synthase complex under threshold Ca^2+^concentrations. Thus, the ability of mtCNPase to interact with ANT and ATP synthase indicates its possible involvement in mPTP regulation [33].

The association of mtCNPase with I, V, III and II complexes in Ca^2+^-loaded RBM showed that mtCNPase could exist in a free form and might be released from the mitochondria along with cytochrome *c* and other apoptotic factors. mPTP opening facilitated the release of mtCNPase from RBM, similar to cytochrome *c*, AIF and Endo G release. In addition, under Ca^2+^-overload conditions, 2′,3′-cAMP furthers the upregulated release of mtCNPase, AIF and Endo G, although it did not alter cytochrome *c* release. The correlation between the release of mtCNPase and AIF and Endo G indicates a possible linkage of mtCNPase with the caspase-independent pathway of apoptosis [33].

## 4. Involvement of mtCNPase in the Regulation of Ca^2+^-Induced mPTP Opening

New functions for mtCNPase in the mitochondria have been reported [16]. CNPase activity was discovered in the outer and inner mitochondrial membranes of RLM and in mitochondria in cultured adrenal cells [4,7]. To investigate whether mtCNPase activity might be changed during the Ca^2+^-induced mPTP opening, an enzymatic mtCNPase activity assay on nitrocellulose membranes was used [38]. Interestingly, the enzymatic activity of mtCNPase in RBM was reduced by 50% underthe Ca^2+^-induced mPTP opening [16]. However, the levels of mtCNPase protein detected before and after mPTP opening were unchanged [16]. The finding that mtCNPase activity in RBM decreased under Ca^2+^-induced mPTP opening provided additional evidence for CNPase working in the mitochondria. The functional importance of CNPase in mitochondria was then unequivocally confirmed by RNA interference experiments using the oligodendrocyte cell line OLN93 [16]. The functional state of mitochondria isolated from wild-type OLN93 cells, scrambled siRNA-treated cells, and cells transfected with siRNA targeting CNPase was measured [16]. The study of the parameters of mitochondria isolated from CNPase knock-down cells revealed that the reduction in CNPase expression facilitates Ca^2+^-induced mPTP opening in the mitochondria from these cells. Table 1 summarizes these results.

No noticeable changes in Ca^2+^ influx rates were observed in mitochondria isolated from various cell types. However, reduced Ca^2+^ capacity (approximately 30%) and lag-phase (approximately 40%) were found for CNPase knock-down mitochondria [16]. Thus, the level of mtCNPase protein in the mitochondria appears to be important for the regulation of Ca^2+^-induced mPTP development.

CNPase hydrolyzes 2’, 3’-cyclic nucleotides to their corresponding monophosphates [1]. The influence of the CNPase substrates, 2′,3′-cyclic nucleotides, on mitochondrial function has also been reported by us [16]. 2′,3′-cAMP and 2′,3′-cNADP significantly enhanced the Ca^2+^-induced opening of mPTP. This effect was observed with Ca^2+^ transport, membrane potential dissipation, and RBM swelling. Both CNPase substrates were able to reduce lag-phase and the increase the rate of Ca^2+^ outflow from RBM under mPTP opening induced by Ca^2+^ [16]. The enzymatic activity of mtCNPase was decreased under Ca^2+^-induced mPTP opening and the hydrolysis of 2’,3’-cyclic nucleotides was prevented [16]. Consequently, the effectiveness of the 2′,3′-cAMP and 2′,3′-cNADP actions on stimulation of mPTP opening by Ca^2+^ -induction in the feedback loop was increased (Figure 2). Therefore, it was proposed that, in living cells, the inhibition of mtCNPase activity under Ca^2+^-induced mPTP opening in the mitochondria contributes to elevating 2′,3′-cyclic nucleotide levels. Thus, CNPase substrates, like 2′,3′-cAMP, seem to work as second messengers by promoting mitochondrial mPTP opening [16].

## 5. CNPase in Pathology and Aging

### 5.1. CNPase in Pathology and Aging

Since CNPase was first detected in myelin, the main study of CNPase was aimed at clarifying its function in the central nervous system. Therefore, to date, there is a large body of work on the role of CNPase in the pathogenesis of diseases of the nervous system. It is known that the destruction of myelin membranes occurs with age, caused not only by oxidative stress and nitration of myelin proteins, but also by the increased activity of calpain and other proteases [39]. Studies conducted on transgenic and knockout mice confirm that CNPase participates in the assembly and formation of myelin membranes and in maintaining the integrity of axons [40,41,42,43]. Thus, CNPase is a potential target for degenerative changes occurring in nerve tissues. It has also been shown that, in the white matter of monkey brain, the content of intact myelin proteins myelin basic protein (MBP) and myelin/oligodendrocyte-specific protein (MOSP) and CNPase are increased [44]. Yang and coauthors showed that the expression of CNPase is increased in activated microglia and, consequently, CNPase knock-down resulted in the enhanced expression of different inflammatory mediators [45]. Chronic microglia activation is known to damage neurons by releasing potentially cytotoxic molecules such as pro-inflammatory cytokines, nitric oxide (NO) and reactive oxygen species (ROS) [46], which can lead to the development of many neurodegenerative diseases [47,48,49]. Thus, CNPase is seen as a potential target for neuroprotection in microglia-initiated neuronal disorders [45].

Increased cognitive deficits, which may be associated with the vulnerability of myelin nerve fibers, occurs with normal aging. It is also known that with brain aging, age-dependent changes happen in key myelin proteins, including CNPase. This leads, in particular, to impaired cognitive function [50]. Loss of nerve fibers and changes in the structure of myelin lead to the ineffective conduction of neuronal signals during aging. CNPase, one of the most common myelin proteins, may play an important role in the maintenance of myelin and axon integrity with age [51].

The previous discussion related only to the participation of myelin-associated CNPase in mechanisms underlying aging and central nervous system pathologies. However, in recent years numerous studies have appeared showing that CNPase present in unmyelinated tissues and cells also plays important roles in the pathogenesis of various diseases, particularly via the mitochondria, which is discussed in the next section.

### 5.2. mtCNPase in Aging

**Figure 3:**
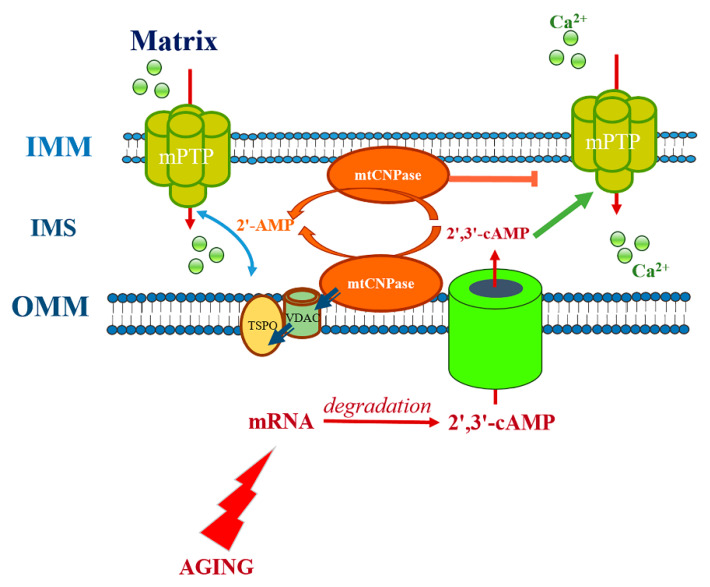
Role of CNPase in mitochondria in aging.

Aging is a process accompanied by progressive mitochondrial dysfunction associated with a continuous decrease in their capacity to produce ATP. In this chapter of the review, we will discuss whether the levels of mtCNPase change mitochondrial fractions with the aging process (Figure 3). Interestingly, the level of mtCNPase was decreased in RBM with aging [52]. Lower mtCNPase content was accompanied by the decreased enzymatic activity of mtCNPase in RBM isolated from the brain of old rats. As we reported in Section 3, mtCNPase co-localized to ADAP1 and VDAC in mitochondria [26,33]. Therefore, the levels of these proteins in RBM isolated from young and old rats and the correlation of mtCNPase levels with the levels of ADAP1 and VDAC in these fractions were compared. The analysis of data showed that both ADAP1 and VDAC levels were lower in RBM isolated from old rats relative to RBM from young animals [52]. The reduced expression of mtCNPase in mitochondria isolated from CNPase knock-down oligodendrocytes (OLN93) resulted in the decrease in the threshold Ca^2+^ concentrations and an acceleration of mPTP opening [16]. Thus, whether there was a link between the alteration in mtCNPase levels in the mitochondria and the induction of mPTP opening with aging was examined. RBM from old rats with lower levels of mtCNPase were more susceptible to Ca^2+^-induced mPTP activation [52]. These results suggest that the age-dependent acceleration of mPTP opening might generally promote the enhancement of susceptibility to cell damage during the aging process. The idea that mtCNPase may participate in the aging process was supported by the finding of accelerated mPTP opening in both young and old animals in the presence of the CNPase substrate 2′,3′–cAMP. Thus, the reduced levels and activity of mtCNPase with aging might diminish 2′,3′–cAMP hydrolysis, thus increasing 2′,3′–cAMP levels, and thereby promoting mPTP opening, which may increase cell damage. The reduction in mtCNPase activity under threshold Ca^2+^ concentrations might further facilitate mPTP opening. The enzymatic activity of mtCNPase was slightly reduced after threshold Ca^2+^-loading in RBM isolated from young rats, but not changed in RBM of old rats, suggesting that there are age-related changes leading to the dysregulation of mPTP opening.

The levels of mtCNPase and ADAP1, and probably VDAC, and their interaction might be essential for age-dependent disorders. Therefore, mtCNPase and ADAP1 are probably signaling molecules involved in the regulation of aging processes. In addition, while the levels of mtCNPase, ADAP1, and VDAC diminish in RBM, they are associated with the Ca^2+^-transporting processes in mitochondria and the regulation of mPTP functioning in aging. The decreased content of these proteins in RBM isolated from old rats correlated with mitochondrial dysfunction [52]. This suggests that the induction of mPTP opening might be the initial stage of mitochondrial dysfunction in general. The endogenous expression of the 2′,3′–cAMP–adenosine pathway has been demonstrated in vivo in both mice and human brain [53]. Experiments with CNPase knockout mice indicated that CNPase was involved in the metabolism of endogenous 2′,3′–cAMP to 2′–AMP and further to adenosine. Traumatic brain injury activated the 2′,3′–cAMP–adenosine pathway in mouse brain. The levels of 2′,3′–cAMP were also increased in the cerebrospinal fluid of patients with traumatic brain injury. Since 2′,3′–cAMP is formed in the cells from mRNA degradation [54], its biosynthesis is therefore stimulated by cell injury [55]. In addition, these data were supported by the finding that brain damage was worsened in CNPase−/− mice after trauma. It is known that the intracellular accumulation of 2′,3′–cAMP is a pro-apoptotic signal via the functioning of mPTP [16,52]. However, the extracellular adenosine is neuroprotective, and consequently, the 2′,3′–cAMP–adenosine pathway is possibly neuroprotective. When cells are damaged, 2’,3’-cAMP metabolism is perhaps used as a source of extracellular adenosine and a critical mechanism for getting rid of the dangerous intracellular 2’,3’–cAMP [53,56]. The reduced expression of mtCNPase in RBM with aging then makes the cells much more susceptible to mPTP opening and likely diminishes the adenosine content, consequently resulting in damage in the interaction of myelin axon. Therefore, mtCNPase plays an important role in the protection of mitochondria from the deleterious effects of the increased intracellular content of 2′,3′–cAMP, which can occur when cells are injured, but such protection may decline with aging as mtCNPase levels fall.

### 5.3. CNPase and Cancer

As mentioned earlier, CNPase is involved in the development of inflammation in neuronal cells [45], and chronic infections and inflammation are considered A risk factors for the development of cancer [57]. On the other hand, mtCNPase participates in the functioning of mPTP and thus is assumed to play a role in apoptosis [16]; therefore, it would be interesting to assess what role this protein plays in the development of different tumors.

Not surprisingly, there are a lot of data showing the presence of CNPase in tumor cells of the central nervous system [58]. CNPase activity was detected in various tumors, such as oligodendroglioma, neurinoma, astrocytoma, undifferentiated gliomas, glioblastoma multiform, medulloblastoma and meningiomas, but activity was noticeably lower than in normal tissues [59]. The presence of CNPase in glioma 6 cells, N1E-115 neuroblastoma [60], and in the HL-60 acute promyelocytic leukemia cell line was noticed [61]. Unfortunately, to date there is no unambiguous understanding of the role of CNPase in the processes occurring during the development of cancer cells, since the data obtained by different groups are contradictory. Zorniak and coauthors observed that the CNPase expression in the xenografts of mouse glioblastoma stem cell and the human tumor samples of glioblastoma multiform correlated significantly with reduced infiltration and improved patient survival [62]. However, according to our results with the neuroblastoma N1E-115 cell clone C-1300, there was no clear correlation between the levels of CNPase expression and differentiation [60]. On the other hand, one unambiguous trend was the increased expression of CNPase during differentiation after the first day of cell culture and the suppression of expression of the protein with increased differentiation after the fourth day of cell culture, under the influence of various factors. An increase in the level of CNPase on the fourth day of culture could be due to the accumulation of unfavorable factors in the medium and the need to increase protection against these factors, which contributed to the activation of CNPase expression as a mechanism for blocking the opening of mPTP and, accordingly, increasing cell resistance [60]. The involvement of CNPase in the processes of THE differentiation and proliferation of mouse neuroblastoma cells was suggested, but this role is probably not determined by the levels of CNPase itself, but rather depends on other environmental factors.

In another study, the increased expression of CNPase in acute myeloid leukemia HL-60 cells in the presence of retinoic acid, an agent used in therapy of this type of cancer, was noticed [61]. Moreover, a correlation was found with decreased expression of other proteins that are components of mitochondrial membranes and regulators of mPTP, VDAC and the translocator protein (TSPO) [61]. TSPO was reported to increase in different types of tumors, including brain tumors and gliomas [63,64,65]. Other reports suggest a key role for TSPO in the development of cancerous tumors [63,66,67,68]. The observed correlation between the expression levels of mtCNPase and TSPO suggests a common mechanism in carcinogenesis.

This suggestion was confirmed during our experiments with TSPO-knockdown glioma c6 cells (unpublished data). The reduction in TSPO expression was accompanied by increased levels of mtCNPase in TSPO knockdown mitochondria. Additionally, we examined whether reduced TSPO expression could influence the previously observed association of mtCNPase with mitochondrial Complexes I-V [33]. TSPO knockdown of glioma C6 cells increased the association of mtCNPase with all Complexes 1.5- to 2.8-fold. This strong association of mtCNPase with the mitochondrial Complexes led us to propose the possible involvement of mtCNPase in the synthesis of some subunits of the Complexes. The mitochondrial genome is packaged into nucleoids, which can be linked to proteins at the inner membrane [69]. Taking into consideration the RNA-binding properties of CNPase [70], it was proposed that the interaction of mtCNPase with Complexes (I-V) could be related to protein synthesis in mitochondria and to RNA processing. The increased expression of mtCNPase, related to the lowering of TSPO expression and the changing of mtCNPase association with Complexes I-V as a result of TSPO knockdown, indicates the functional linkage of TSPO to mtCNPase. Based on these observations, finding the link between TSPO and mtCNPase expression may be important; it could indicate that mtCNPase is involved in protein synthesis in the mitochondria and might even regulate the levels of the mitochondrial pool of TSPO, although this protein is encoded by nuclear DNA. Alterations in mtCNPase-based protein synthesis, then, could be involved in carcinogenesis. It should be noted that although there are currently no unequivocal opinions on the role of mtCNPase in carcinogenesis, our studies strongly imply its involvement in the processes of tumor development.

### 5.4. mtCNPase and Acute Heart Failure

Since the mitochondria are the most important organelle for normal functioning of the heart, disruption of the functional state of mitochondria is a frequent cause for the development of disorders of the cardiovascular system [71]. ATP production, the maintenance of C_a_^2+^ homeostasis and the permeability of both inner and outer mitochondrial membranes are the most important characteristics of mitochondrial function; therefore, disturbances in these processes could lead to pathologies. Thus, an aberration in ATP synthesis in mitochondria, mediated by changes in calcium transport, has been observed in metabolic heart disease. The decreased production of ATP and retractile store in the myocardium in metabolic heart disease have been observed [72]. Mitochondrial C_a_^2+^ is also essential to the production of ROS and the functioning of mPTP. In the processes of both ischemia/reperfusion and heart failure, these factors are involved [73,74,75].

Cell death after such disturbances due to mitochondrial intrinsic killing mechanisms, necrosis and apoptosis, underlies a host of cardiac diseases [76]. By regulating the permeability of its membranes, mitochondria control necrotic and apoptotic myocardial cell death [77]. Thus, the permeabilization of outer membranes by proteins of the Bcl-2 family and mPTP regulation of the permeability of inner membrane may be considered as a central events in necrosis and apoptosis [77]. The role of mPTP in several cardiovascular diseases has been widely discussed [78,79,80,81,82]. The evidence suggests that mPTP participates in cardioprotective signaling pathways either directly or indirectly. Thus, mPTP has become a promising drug target in therapeutic approaches. However, despite many years of research, the complexity of process has stymied researchers, and the molecular identity and the mechanism of regulation of mPTP in the heart is still unclear.

We have shown that mtCNPase participates in the functioning of mPTP and CyP-D, VDAC, ANT, and α-tubulin co-localized with it [33]. Since mPTP inhibition is considered an important criterion for protecting the heart against heart disturbance and heart failure, the search for targets for controlling the mechanisms of mPTP opening is the most important task for finding new therapeutic strategies based on mPTP. The use of antioxidants that can reduce oxidative damage and, as a result, enhance the protective reaction and prevent the development of mitochondrial damage seems very promising. There are many oxidizable substrates in the mitochondria, including proteins, lipids, carbohydrates, and DNA, that could be damaged by oxidative stress. There are a number of both synthetic and natural antioxidants with various mechanisms of action. In connection with the assumed role of oxidative stress in cardiovascular diseases, clinical studies have been conducted on the effect of various antioxidants, including vitamin E, on the development of these diseases [83]. However, these studies did not show a significant positive effect with any of the antioxidants used, which is probably due to the inability of some antioxidants to accumulate in the mitochondria, the main site of ROS production. There are a number of antioxidants that can directly affect the mitochondria, which certainly makes them promising therapeutic agents for mitochondrial-targeted treatment strategies [83,84,85,86].

One such antioxidant is melatonin (MEL), a hormone synthesized by the pineal gland. MEL is a lipophilic molecule, and therefore easily penetrates through membranes, reaching subcellular structures [87]. Since we previously showed a relationship between the protective action of MEL on the mitochondria during aging and the decreased levels of mitochondrial mtCNPase with age [88,89], we suggested the presence of a distinctive mechanism for MEL action in mitochondria in relation to pathologies in which mtCNPase may be involved as a key player [90]. This hypothesis was confirmed in experiments on the effect of MEL on the functional state of rat heart mitochondria (RHM) isolated from rats with acute heart failure caused by the isoprenaline hydrochloride injection [91]. The results showed that the levels of mtCNPase increased in RHM isolated from rats with acute heart failure, probably to protect them from damage, and that MEL administration strengthened this effect. The reduced levels of VDAC in RHM from rats with acute heart failure was observed, but MEL did not abolish the effect. Because VDAC and mtCNPase co-precipitated [33], it was proposed that mtCNPase might compensate for VDAC regulation in the mitochondria. The detailed mechanism of this phenomenon remains to be investigated; however, the role of mitochondrial mtCNPase in cardioprotection is undeniable [91].

In addition, the effect of astaxanthin (AST) on the parameters of mitochondrial function was investigated. AST, as an antioxidant, has anti-inflammatory properties and potential as a therapeutic agent for many heart diseases. AST is a xanthophyll carotenoid, a red pigment, giving sea animals, such as salmon, trout, common shrimp, and lobster, their distinctive reddish coloration [92]. There are plants, algae and microorganisms that contain a high concentration of such carotenoids, which are rather ubiquitous. Humans cannot synthesize them and therefore are required to source them in their food intake [93]. AST contains two oxygenated groups on two ring structures, which determines its enhanced antioxidant abilities [94]. In a model of homocysteine-induced cardiotoxicity, AST was able to restore the integrity of the mitochondria and inhibit mitochondrion-mediated apoptosis [95,96]. The addition of AST prevented the opening of mPTP, and thus improved mitochondrial function and reduced the sensitivity of mitochondria to stress.

AST treatment also reduced the levels of TSPO, VDAC, and mtCNPase, the proteins participating in regulation of mPTP in native RHM [97]. Localized to the intermembrane space side of the outer membrane, mtCNPase can be associated with VDAC and regulate the permeability of the outer mitochondrial membrane and influence the conductance of the VDAC channel. [33]. It has been suggested that the decreased level of VDAC in RHM in AST-pretreated animals may cause a change in Ca^2+^ sensitivity in these animals [97]. It is known that AST can activate the cAMP/PKA/CREB signaling system in the brain [98], and the results of our research have shown that mtCNPase can be phosphorylated by PKA [99]. In addition, it was found that AST is able to reduce the levels of CREB phosphorylation (cAMP-responsive element-binding protein) in mitochondria. CREB activation causes mitochondrial function impairment [100]. To summarize, AST can inhibit the induction of mPTP opening and change the levels of mPTP regulatory proteins by both the direct addition of AST to isolated mitochondria and mitochondria isolated after the administration of AST to rats, increasing the resistance of RHM to Ca^2+^-dependent stress. It has previously been suggested that mtCNPase could be the target of the anti-apoptotic effects of AST in the mitochondria [97]. In another case, the effect of the administration of AST on ISO-induced impairment of RHM was observed [101]. In RHM from rats pretreated with AST, the levels of mtCNPase decreased, whereas subsequent ISO injection resulted in an increase in the content of mtCNPase. The content of mtCNPase was also increased by administration of AST in combination with ISO, relative to the control and the effect of ISO alone. Recently, we showed that mtCNPase in RBM was associated with each complex of ETC and ATP synthase [33], however, in the work with RHM [101], it was found that mtCNPase is associated only with CIII, implying that this connection is tissue-specific.

**Figure 4:**
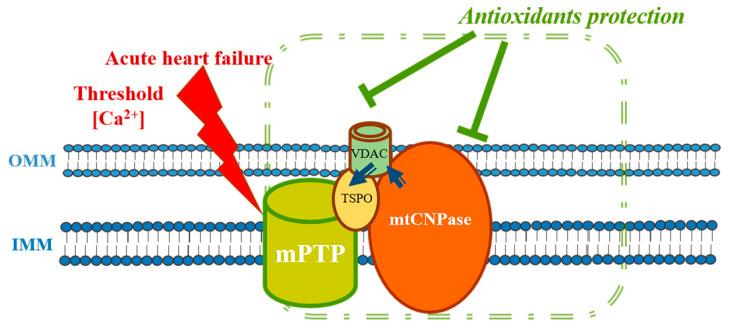
Role of CNPase in the development of acute heart failure.

It has been proposed that mtCNPase fulfills a protective function and can be a target for the effect of AST in RHM. This hypothesis will be verified in experiments of chronic administration of AST on rats with acute heart failure in the future. Nevertheless, AST can be considered as an effective drug to improve cardiac function under normal and pathological conditions and its molecular target seems to be mtCNPase. Figure 4 summarizes the role of mtCNPase in the development of acute heart failure.

### 5.5. mtCNPase and Alcohol

There are considerable data about the key role of mitochondria in the pathogenesis of diseases associated with alcohol consumption [102]. Moreover, both chronic and acute alcohol poisoning affect the operation of mitochondria, disrupting normal function [103,104,105]. Chronic alcohol consumption leads to multiple liver impairments, including steatosis, which can progress with continued alcohol consumption and lead to the formation of alcoholic steatohepatitis, liver fibrosis, cirrhosis and other liver diseases that ultimately lead to hepatocellular cancer [106,107,108]. Despite intensive research, the factors that link ethanol consumption to the onset and progression of liver disease remain debatable [109,110,111,112,113].

In chronic alcohol consumption, substantial changes in the mitochondria do occur [114,115]. Ethanol-mediated changes include abnormalities in mitochondrial morphology (for example, enlarged, deformed mitochondria with fewer cristae appear) [116] and the increased production of ROS [109]. The study of mitochondria in chronic alcohol consumption is important because alcohol affects the redox signaling pathways and acts as a regulator of cellular Ca^2+^ homeostasis [117,118]. Changes in the regulation of Ca^2+^ homeostasis are involved in the mechanisms of cell death [119]. Studies in various laboratories showed that the chronic consumption of ethanol initiates the formation of the mPTP [120,121,122]. In this regard, it seems promising to consider the participants and regulators of mPTP as potential targets in developing strategies for treating the effects of alcohol abuse. As mentioned earlier, despite years of research, the exact composition of the pores has not yet been established. However, there are a number of proteins that are considered as pore regulators [16,27,123,124], including mtCNPase.

mtCNPase is one of the most promising potential targets among the many pore regulators. There are studies showing the participation of proteins with phosphodiesterase activity in the mechanisms of disorders associated with alcohol consumption [125]. As a critical regulator of intracellular and intramitochondrial cAMP levels, phosphodiesterases are able to take part in the regulation of pathological processes of alcohol exposure [126]. Our preliminary data showed the participation of mitochondrial mtCNPase in the regulation of mPTP under the conditions of chronic alcohol consumption. A decrease in the expression of mtCNPase in RLM with chronic alcohol intoxication, as well as changes in the levels of tyrosine kinase phosphorylation that is involved in the phosphorylation of mtCNPase itself, were observed. Taken together, our preliminary results and published data suggest that mtCNPase is a promising new target for drugs treating the effects of alcohol dependence.

## 6. Conclusions

**Figure 5:**
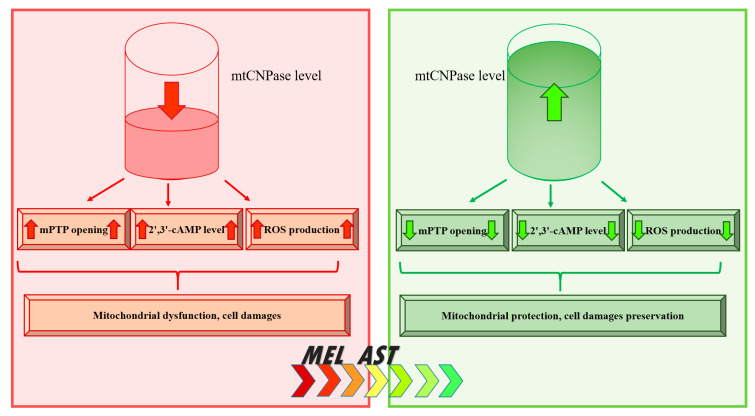
Schematic representation of the role of CNPase in pathologies.

In this review, the main functions of mtCNPase, independent of myelin structures, are evaluated. We reviewed the basic aspects of its detection in mitochondria and showed its participation in the regulation of mPTP functioning. mtCNPase interacts with proteins such as ANT, VDAC, CyP-D and thereby is capable of controlling mPTP functioning. mtCNPase is associated with respiratory chain complexes, and mitochondrial ATP synthase, which can lead to the release of mtCNPase from mitochondria in parallel with the release of cytochrome *c*, AIF, and Endo G. In addition, based on a study of the role of mtCNPase in pathological conditions, such as aging, heart failure, cancer, and alcohol addiction, we propose a scheme outlining the role of mtCNPase in these processes (Figure 5). In mitochondria, the decrease in the mtCNPase level leads to the acceleration of mPTP opening, accompanoed by accumulation of the 2′,3′-cAMP, the increase in ROS production, and consequently, mitochondrial dysfunction and cell injury. The increase in the mtCNPase level results in the inhibition of mPTP opening, the decrease in the content of 2′,3′-cAMP and ROS production that, in turn, leads to protect cell against damage. Antioxidants, such as MEL and AST, under mitochondrial impairment conditions, enhanced the levels of mtCNPase, thereby, suggesting a protective effect for the protein in mitochondria and increasing mitochondrial efficiency. Although the mechanisms of these phenomena are not fully understood and require further research, mtCNPase could be considered a potential target in the development of therapeutic strategies for the prevention of diseases of various etiologies.

## Figures and Tables

**Table 1:** Influence of CNPase knock-down on mitochondrial functional parameters at Ca^2+^-induced mPTP opening.

	Relative Values of Ca^2+^ Influx Rate	Relative Values of Lag Time	Relative Values of Ca^2+^ Capacity
Wild type	1.00 ± 0.11	1.00 ± 0.06	1.00 ± 0.14
Scrambled siRNA	0.97 ± 0.10	0.85 ± 0.08	0.98 ± 0.05
CNPase siRNA	0.87 ± 0.13	0.66 ± 0.09 **	0.68 ± 0.11 *

* *p* < 0.05; ** *p* < 0.001.
